# Grant application outcomes for biomedical researchers who participated in the National Research Mentoring Network’s Grant Writing Coaching Programs

**DOI:** 10.1371/journal.pone.0241851

**Published:** 2020-11-09

**Authors:** Anne Marie Weber-Main, Richard McGee, Kristin Eide Boman, Japera Hemming, Meldra Hall, Thaddeus Unold, Eileen M. Harwood, Laurie E. Risner, Ann Smith, Kimberly Lawson, Jeffrey Engler, Clifford J. Steer, Dedra Buchwald, Harlan P. Jones, Spero M. Manson, Elizabeth Ofili, Nancy B. Schwartz, Jamboor K. Vishwanatha, Kolawole S. Okuyemi

**Affiliations:** 1 Department of Medicine, University of Minnesota Medical School, Minneapolis, Minnesota, United States of America; 2 Department of Medical Education, Northwestern University Feinberg School of Medicine, Chicago, Illinois, United States of America; 3 Department of Family and Preventive Medicine, University of Utah School of Medicine, Salt Lake City, Utah, United States of America; 4 Clinical Research Center, Morehouse School of Medicine, Atlanta, Georgia, United States of America; 5 Division of Epidemiology and Community Health, University of Minnesota School of Public Health, Minneapolis, Minnesota, United States of America; 6 Department of Pediatrics, University of Chicago, Chicago, Illinois, United States of America; 7 Department of Medicine, University of Alabama at Birmingham, Birmingham, Alabama, United States of America; 8 Council of Graduate Schools, Washington, District of Columbia, United States of America; 9 Department of Medicine and Department of Genetics, Cell Biology, and Development, University of Minnesota Medical School, Minneapolis, Minnesota, United States of America; 10 Institute for Research and Education to Advance Community Health, Elson S. Floyd College of Medicine, Washington State University, Spokane, Washington, United States of America; 11 Center for Diversity and International Programs, University of North Texas Health Science Center, Fort Worth, Texas, United States of America; 12 Colorado School of Public Health, University of Colorado Anschutz Medical Campus, Aurora, Colorado, United States of America; 13 Department of Medicine, Morehouse School of Medicine, Atlanta, Georgia, United States of America; 14 Department of Pediatrics and Department of Biochemistry and Molecular Biology, University of Chicago, Chicago, Illinois, United States of America; Charles P. Darby Children's Research Institute, UNITED STATES

## Abstract

**Background:**

A diverse research workforce is essential for catalyzing biomedical advancements, but this workforce goal is hindered by persistent sex and racial/ethnic disparities among investigators receiving research grants from the National Institutes of Health (NIH). In response, the NIH-funded National Research Mentoring Network implemented a Grant Writing Coaching Program (GCP) to provide diverse cohorts of early-career investigators across the United States with intensive coaching throughout the proposal development process. We evaluated the GCP’s national reach and short-term impact on participants’ proposal submissions and funding outcomes.

**Methods:**

The GCP was delivered as six similar but distinct models. All models began with an in-person group session, followed by a series of coaching sessions over 4 to 12 months. Participants were surveyed at 6-, 12- and 18-months after program completion to assess proposal outcomes (submissions, awards). Self-reported data were verified and supplemented by searches of public repositories of awarded grants when available. Submission and award rates were derived from counts of participants who submitted or were awarded at least one grant proposal in a category (NIH, other federal, non-federal).

**Results:**

From June 2015 through March 2019, 545 investigators (67% female, 61% under-represented racial/ethnic minority, URM) from 187 different institutions participated in the GCP. Among them, 324 (59% of participants) submitted at least one grant application and 134 (41% of submitters) received funding. A total of 164 grants were awarded, the majority being from the NIH (93, 56%). Of the 74 R01 (or similar) NIH research proposals submitted by GCP participants, 16 have been funded thus far (56% to URM, 75% to women). This 22% award rate exceeded the 2016–2018 NIH success rates for new R01s.

**Conclusion:**

Inter- and intra-institutional grant writing coaching groups are a feasible and effective approach to supporting the grant acquisition efforts of early-career biomedical investigators, including women and those from URM groups.

## Introduction

A diverse research workforce is essential for identifying and finding solutions to complex biological problems that will lead to medical breakthroughs and improve human health [1]. Across various training and professional settings, diversity yields higher performance in problem solving situations, greater innovation and creativity, and enhanced productivity and performance [2–7]. Within biomedical research fields (inclusive of biomedical, behavioral, clinical, and social sciences), workforce diversity can expand the range of questions that are addressed and prioritized, including those focused on reducing health disparities or other community-driven needs [8–12]. Heterogeneity in scientific teams may even contribute to science quality and publication impact [13,14]. Thus, it is of critical public health importance that individuals from underrepresented groups are trained in biomedical disciplines and supported in developing successful research careers.

Despite the U.S. becoming more racially and ethnically diverse, the proportion of investigators from underrepresented racial and ethnic minority groups (URM) who receive major research grants from the National Institutes of Health (NIH) remains unacceptably low [15,16]. An examination of fiscal years 2000 to 2006 found that compared with NIH R01 applications from white investigators, those from black investigators were 13 percentage points less likely to be awarded [17]. Applications from other URM groups such as American Indian/Alaska Native investigators were too few to even include in those analyses. Similar disparities by race/ethnicity and sex were reported for 2010 to 2013, with R01 applications from black and female investigators less likely to be funded than those from white or male PIs [18]. Despite growth from 2002 to 2016 in the number of female and URM investigators who applied for and were awarded NIH grants, gaps in the R01 funding rate by race/ethnicity and sex have persisted. In 2016, this gap was 7.5 percentage points between white and URM applicants, and 2.1 percentage points between men and women applicants [15].

Recognizing the urgency to bridge these gaps and fully realize the benefits that diversity offers, the NIH established the Diversity Program Consortium [19]. This trans-NIH program is a national collaborative of U.S. higher education and research institutions working in partnership with the NIH to enhance the participation and persistence of individuals from underrepresented backgrounds in biomedical research careers. One of the Consortium’s core components is the National Research Mentoring Network (NRMN) [20]. During phase I of NRMN implementation (2014–2019), a variety of initiatives were launched to improve the quality and accessibility of research mentoring and to support the professional development of individuals at different stages of the training/career pipeline.

This article describes early outcomes for one NRMN phase I initiative, the Grant Writing Coaching Programs (GCPs). The GCPs were designed to provide diverse cohorts of early-career investigators (predominantly postdoctoral fellows and junior faculty) with sustained and intensive coaching in grant proposal development over 4 to 12 months [21]. Four, later expanding to six, different NRMN GCP models were implemented. Although the models differed slightly in their target audience, duration, and programming, all operated with a shared commitment to engage early-career investigators from underrepresented groups; to reach beyond institutional boundaries to benefit those with limited access to local mentorship; and to support investigators at critical transition points (e.g., from postdoctoral training to faculty positions, from pilot funding to larger awards, from mentored trainee to independent investigator). Further, the models shared the core intervention approach of enrolling participants as a cohort to facilitate peer mentorship and connecting them with experienced investigators (grant writing coaches) for iterative feedback on their in-progress grant applications over several months of development.

Here, we report results documenting the reach and near-term impact of the NRMN GCPs from June 2015 (when the first coaching groups were initiated) through March 31, 2019. First, we present demographic characteristics of the more than 500 individuals who participated in a GCP, including the proportion of women and URMs and their distribution by institution type (Carnegie classification) and geographic location. Second, we report summary statistics on the number and type of grant applications submitted by and awarded to participants within the first 18 months of GCP completion. Third, we present submission rates (number of unique participants who submitted at least one proposal/total number of participants) and award rates (number of unique participants who were awarded at least one grant/total number of submitters) for different categories of grant applications: applications to the NIH, to other federal agencies, and to non-federal sources. Outcome metrics are reported for the full sample and participant subgroups by gender, URM status, and GCP model.

## Methods

This study was reviewed and granted exempt status by the Institutional Review Boards of the University of Minnesota, University of Utah, and University of Chicago.

### Description of the GCPs

The overarching design for all of the NRMN GCPs was based on the same core premise: Grant writing is a complex but teachable skill that is best acquired through repetitive cycles of practice over a sustained period of active project development, during which improvement is driven by the input of highly skilled practitioners (grant writing coaches).

In NRMN year 1, four coaching models–each with a recognized record of successful training, either on a national scale or within individual institutions–were adapted for national implementation through the network. These models were as follows: STAR—Steps Towards Academic Research, developed at the University of North Texas Health Sciences Center; GUMSHOE—Grant writing Uncovered: Maximizing Strategies, Help, Opportunities, and Experiences, developed at the University of Colorado Anschutz Medical Campus and Washington State University; NU—the Northwestern University Grant Writers Coaching Groups; and P3—the Proposal Preparation Program, developed at the University of Minnesota. Descriptions of these four models have been published previously [21].

After year 1, two variations of the NU model were introduced to reach specific target audiences. The first was SETH (Strategic Empowerment Tailored for Health Equity Investigators), developed at Morehouse School of Medicine [22]. SETH predominantly enrolled investigators working at collaborating institutions funded by the NIH’s Research Centers in Minority Institutions (RCMI) network, Institutional Development Award (IDeA) network, and Clinical and Translational Science Awards (CTSA) program. The second variation was CAN (Committee on Institutional Cooperation Academic Network), developed at the University of Chicago and funded by a supplement grant from the NRMN [23]. CAN invited its participants from member universities of the Big Ten Academic Alliance, with a focus on enhancing the faculty-readiness of URM postdoctoral fellows (although junior faculty were also included when appropriate).

Essential curricular elements of all six programs are provided next to facilitate their replication or adaptation by others and to contextualize the outcomes that we present later in this article.

#### Shared core curriculum

**Fig 1:**
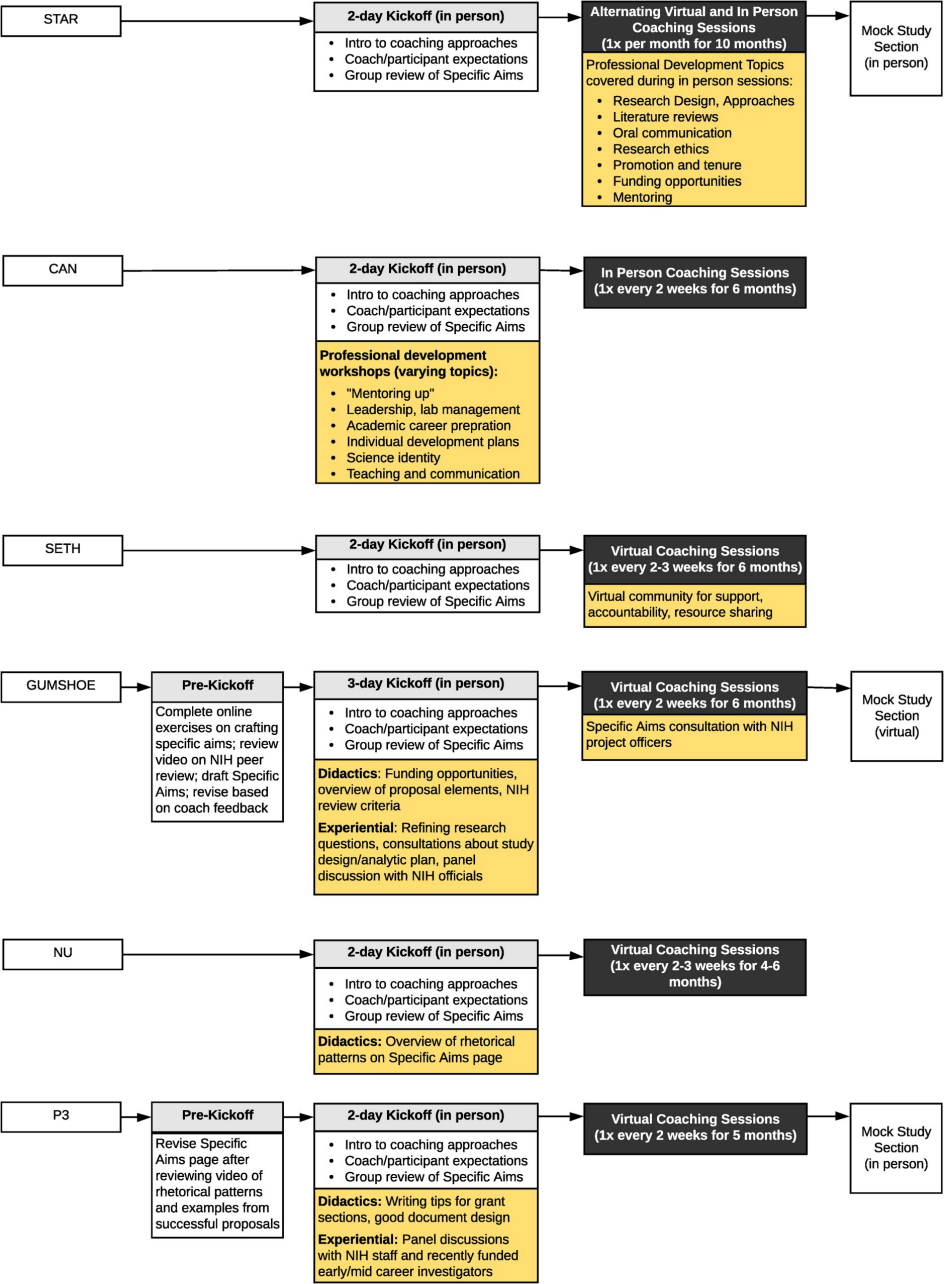
Core curricular elements of the NRMN Grant Writing Coaching Program models.

Fig 1 describes the core elements of the six GCP models. All models began with an intensive, 2- or 3-day in-person kickoff session (held on the campus of each model’s founding institution or identified partners). This session served three important purposes. First, it gave participants and their grant writing coaches the opportunity to meet face-to-face in an informal setting and begin learning about one another’s research interests, institutional environments, and past proposal writing experience. Second, program directors communicated their expectations for how the coaching dyads/groups would function and how the program would support their activities. Third and most importantly, coaching dyads/groups began actively working together, participating in breakout sessions to review and discuss participants’ draft Specific Aims pages. This approach to initiating the GCPs was designed to foster a rapid but deep introduction to each participant’s research project while building the rapport that would help the coaching units collaborate productively throughout the subsequent months.

The second curricular element shared by all of the GCP models was a series of grant writing coaching sessions conducted over several months to support participants’ active proposal writing efforts. Coaching occurred in either dyads (GUMSHOE) or small groups (all other models). Coaching sessions were implemented virtually, with a few exceptions. The STAR model used a blend of in-person and virtual sessions. Because CAN participants and coaches were located at Big Ten Academic Alliance institutions, the cohort separated into smaller coaching groups that met in person on their home campuses after the initial kickoff.

Between coaching sessions, participants drafted and revised sections of their proposals. They then met with their assigned coaches as dyads or groups to receive tailored oral feedback on their work in progress. Coaches drew on their deep experience as externally funded biomedical investigators and proposal reviewers to offer constructive critiques, as well as expose their thought processes and candid reactions to drafted text. In this manner, coaches helped participants to deconstruct their writing and make strategic revision plans to address readers’ needs and have the greatest impact. Depending on the model, participants were also encouraged (and in some models expected) to offer feedback on their peers’ drafts. By situating coaching within small peer groups, the GCPs promoted vicarious learning. Participants were able to emulate strong writing examples from other group members, as well as discuss common issues they were encountering during proposal development. Working in groups also fostered accountability and social support.

#### Unique model features

**Fig 2:**
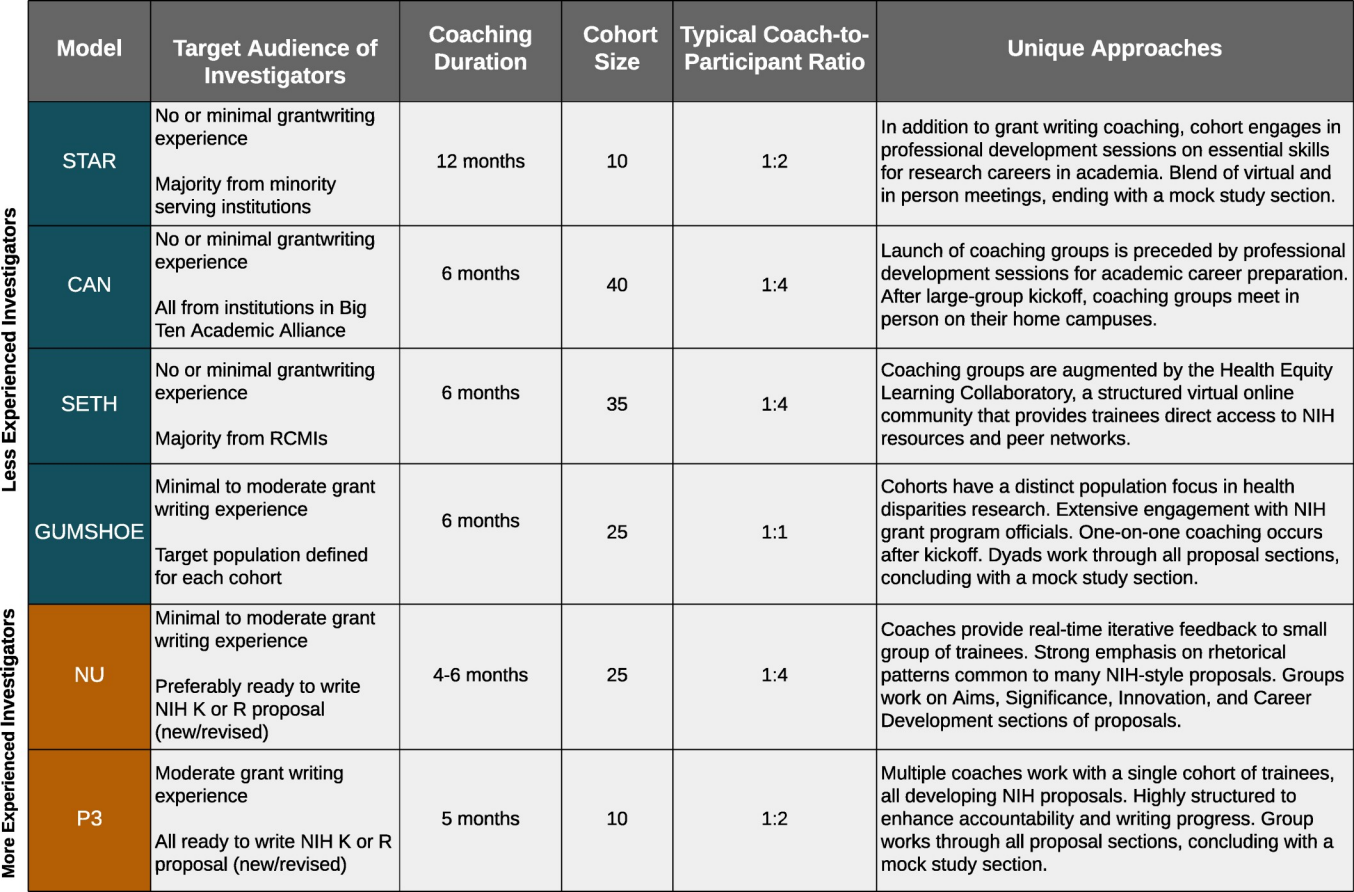
Unique features of the NRMN Grant Writing Coaching Program models.

Although all of the GCP models shared a common core approach to training, each had some intentional differences in their ancillary activities, coaching session frequency, and overall program duration (shown in Figs 1 and 2). Some of these design differences were driven by the background of their target audiences. For example, models that enrolled individuals with less experience in grant writing and research (STAR, CAN, SETH, and GUMSHOE) tended to be longer while also providing supplemental activities and consultations to help participants hone their scientific ideas and expand their knowledge about different funding opportunities. Three of the six models concluded with an NIH-style mock review session.

A few unique features of each GCP are worth highlighting. Both STAR and CAN supported skill building beyond proposal preparation to support their participants’ readiness for and advancement in academic research careers (see Fig 1 for topics). SETH was unique in employing an online Health Equity Learning “Collaboratory.” This virtual community gave participants access to enhanced social support from peers, proposal feedback and writing accountability from coaches, and additional grant preparation resources. GUMSHOE sought to optimize peer-coach interactions by focusing each cohort on research that engaged a specific population, such as Latinos, American Indians and Alaska Natives, or members of rural communities. NU was a “lighter touch” model in that coaching usually did not extend very far into the Approach section of a proposal; however, participants benefited from rich and lengthy oral feedback from NU coaches on other proposal sections. P3 uniquely engaged multiple coaches (4 to 6) working together with the full cohort of 10 to 12 participants. In this design, participants received a diverse range of feedback on their work-in-progress from coaches with different types of expertise. Additionally, P3 imposed more structured guidance for how often the coaching groups would convene and the expected level of progress on the proposals. An example P3 coaching schedule, including specified writing assignments is provided as S1 Fig.

#### Coach qualifications and roles

To achieve national scalability, each GCP recruited experienced investigators to serve as coaches. A total of 119 individuals served as NRMN GCP coaches. They were predominantly mid- to late-career scientists (58% Full Professors, 30% Associate Professors, 3% Assistant Professors); 48% were female, and 42% were from URM groups. Coaches were recruited through the NRMN website, the professional networks of NRMN leaders and members, and open solicitations to scientific societies and groups. For CAN, institution-specific coaches were recruited from universities in the Big Ten Academic Alliance. GUMSHOE also recruited from institutions that co-hosted their in-person kickoff workshops. Individuals applied to be a coach by completing a brief application and submitting an NIH biosketch or curriculum vitae. Materials were reviewed by program directors to assess qualifications. Generally, coaches had to demonstrate several years of successful grant writing experience; a strong track record and/or demonstrated interest in mentoring early-career investigators, particularly those from underrepresented populations; experience serving on grant review panels (preferable); and sufficient time to fulfill the coaching expectations of a particular model. With the exception of CAN whose coaches participated as volunteers, coaches received nominal compensation for their roles.

Coaches’ roles differed somewhat across models. In P3 a team of 4–6 coaches worked together with a group of 10–12 trainees over the 5 months of coaching. Coaches for NU, SETH, and CAN were first introduced to the coaching approach and then assigned to work individually or in pairs with a small group of 3–5 trainees for 4 to 6 months. For GUMSHOE, participants were generally matched with a single coach, with expectations for one-on-one engagement over 6 months. Coaches in STAR typically worked with two investigators for a full year, while participating in additional professional development sessions with the full cohort. At the kickoff session for each model, coaches received a similar orientation to the grant writing coaching process (its value, best practices). This orientation was supplemented with information about the model’s specific coaching expectations and procedures.

### Participant recruitment

Across all models, 35 coaching cohorts were initiated between June 15, 2015 and December 31, 2018. Announcements of program availability, design features, and participant eligibility were distributed nationally by electronic means (email, listserve postings, e-newsletters) to relevant audiences. Target audiences included directors of institutional research training programs, leaders of NIH-funded programs (such as the RCMI network), and members of scientific societies and diversity-focused professional organizations such as NRMN, the Society for Advancement of Chicanos/Hispanics and Native Americans in Science, the Leadership Alliance, and the American Association of Minority Physicians. For later cohorts, past GCP participants and coaches were encouraged to share program information with others in their professional networks. Announcements directed readers to the NRMN website for program descriptions and application links.

The web-based application collected information about applicants’ past scientific activities (such as publications, previous proposal submissions, grant awards), type of proposal they were developing, stage of writing, and accessibility to mentoring and other institutional research support. Applicants provided an NIH-style biosketch and for some programs a draft of their specific aims page. Applicants were required to register with NRMN through its web portal, which collected demographic information on anyone who engaged with NRMN initiatives. A single application was used for the first cohorts of STAR, P3, and NU. Directors of those programs worked together to evaluate applicants and place them in the most appropriate model. In subsequent rounds, individuals applied to each program separately.

### Participant selection

The broad objective in reviewing GCP applications was to identify individuals in need of coaching and ready to benefit from the intensive writing process and professional development activities of a particular model. Program directors employed a holistic review in which no single factor took precedence in selection. For example, need for coaching was evaluated based on factors such as the applicant’s career stage, prior experience in proposal writing, and access to mentors. Readiness to benefit was assessed by considering the applicant’s previous experience and success in grant writing and their project’s current stage of development. Number and type of publications were considered to the degree that they met reviewers’ expectations for the funding mechanism the applicant was targeting. Additional factors such as institutional setting, research resources, and teaching or clinical expectations were also examined.

Some selection criteria differed by program. P3 required that applicants be ‘ready to write’ and aiming for proposal submission within 6 months of program completion. Thus, someone who was still gathering data to determine the core direction of a project or was a year out from submission would not be a strong P3 applicant. In contrast, applicants for the NU and CAN models could still be gathering data and have a more tentative anticipated submission date. STAR and GUMSHOE were designed to accommodate individuals with a longer timeframe for proposal development and submission; both enabled more flexibility in how participants worked with their coaches and progressed in their proposal development. GUMSHOE’s selection process also considered applicants’ commitment to working with the priority population that was defined for each cohort.

Program directors reviewed all applications and would occasionally refer applications to other programs that seemed more appropriate. Applications were ranked, and individuals considered to have the greatest need and ability to benefit were offered a position first. The remaining positions were filled according to rank order. In any application group, a small subset of individuals were rejected because their experience or goals were clearly misaligned with the GCP (e.g., their research interests were well outside the scope of biomedicine and public health; they expressed a vague idea of the research they wanted to pursue; they needed substantially more preliminary work and/or publications to be competitive for their intended grant mechanism; and/or they already had a substantial track record of success in acquiring grants and were well connected to highly skilled and engaged mentors, suggesting little need for coaching). That said, the programs accepted participants with a fairly wide range of past grant writing experience, project development, and readiness-to-write. This diversity was purposeful and valued, as we sought to engage as many early-career investigators as possible and recognized that our ability to predict need and readiness based on relatively brief written applications was modest. In later cohorts, SETH and NU employed short phone or video conversations with applicants to better assess alignment with the program. P3 began requiring applicants to submit the name of a local mentor or experienced colleague who supported the applicant’s readiness for the program and were willing to periodically provide scientific input on the applicant’s proposal. CAN required recommendations from institutional leadership and the approval of PIs for their postdoctoral fellows’ participation.

### Data collection and analyses

Demographic data on GCP participants were obtained from their application and NRMN portal registration. These data included sex (female, male), race/ethnicity (American Indian/Alaska Native, Asian, Black/African American, Hawaiian/Pacific Islander, Hispanic, White, Other, Not specified/Missing), current career stage (postdoctoral fellow, faculty rank, or non-academic research position), education (highest academic degree), and institutional affiliation.

Primary outcomes were number and type of grant applications submitted by and awarded to participants after GCP completion. Data were acquired from online assessment surveys administered through REDCap (a secure web application for building and managing online surveys and databases). Requests for survey completion were sent by email to GCP participants at the end of each program and at 6, 12 and 18 months after program completion. At least two follow-up emails were sent to non-responders. When response rates were low, program directors sent targeted emails to encourage completion. Here, we report data collected from June 15, 2015 through March 31, 2019. For that end date, participants in earlier GCP cohorts would have completed their 18-month assessments and were no longer being followed by NRMN. Others were at earlier phases of data collection. For the majority of participants, the data presented represent outcomes for approximately 24 months after starting a coaching program.

A primary goal of the GCPs was to foster participants’ success in obtaining NIH and other federal-level awards. For these types of applications, we verified and supplemented self-reported data by conducting systematic searches every 6 months of public repositories of federally funded grant applications, including NIH Reporter, Federal Reporter, and Grantome. This strategy was unable to identify awards that did not link directly to the participant, such as NIH Diversity Supplements. For those types of awards, we relied on self reporting.

Data from all GCP models and cohorts were merged into a single project database. Our analyses were based on unique participants; individuals who participated in more than one GCP model or cohort were counted only once, and for analyses were associated with the first program they completed.

We conducted descriptive analyses for the variables of interest (demographics and proposal outcomes) using the numbers and proportions of unique participants per category. We divided grant proposal submissions and awards into three high level categories for analysis: NIH, Other Federal, and Non-Federal (e.g., private foundations, professional scientific associations, state-level grants, institutional awards). NIH proposals were further broken down to distinguish major research awards (R01s and similar mechanisms) from others such as fellowship awards (F series), mentored career development awards (K series), and other research awards (e.g., R03, R21, SC1/2).

We first calculated the total number of grants awarded to GCP participants. For this outcome, multiple awards to the same participant were included. We determined numbers and proportions of awarded grants for the total sample and for participant subgroups–URM and female participants–within each of the grant application categories defined above.

Second, we assessed proposals submitted and grants awarded as counts and proportions of individuals who submitted at least one application or received at least one grant per category. For these analyses, individuals who submitted more than one proposal or received more than one grant in a particular category were counted only once. We adopted this approach to enable a descriptive comparison of submission rates and award rates–overall, and across different funding categories–for the various GCP models and for participant subgroups (by URM status, gender, or Carnegie classification of home institution). Given small cell sizes for some categories and inherent differences in the GCP models, we limited our statistical tests to two separate mixed model logistic regressions. One model tested the effects of URM status on overall proposal submission rates and funding rates, adjusting for clustering by program and cohort. The second model tested the effects of sex on these same outcomes. We performed similar analyses for NIH grants only. We did not test for additive effects of programs.

## Results

### Participant demographics

A total of 573 individuals participated in the GCPs. A small number of participants took part in more than one program or cohort (26 participated twice, 1 participated three times). Thus, the number of unique trainees was 545. Unless otherwise stated, we report results for this sample of unduplicated participants.

As shown in Table 1, 67% of the 545 participants were female and 61% were from racial or ethnic minority groups that are underrepresented in the U.S. biomedical research workforce [24]. The majority were either Assistant Professors (50%) or postdoctoral fellows (34%). Investigators with PhDs were over-represented. Some differences in participant characteristics were observed by program. For example, CAN enrolled the largest proportion of postdoctoral fellows (74%), having been designed to focus predominantly on this training stage. GUMSHOE had the highest participation rate of individuals identifying as American Indian, Alaska Native, or Native Hawaiian-Pacific Islander (14%). The first and fifth cohorts of GUMSHOE intentionally focused on recruiting investigators from and/or working with these populations.

**Table 1:** Characteristics of participants in the NRMN Grant Writing Coaching Program, total sample and by model.

Characteristic	Grant Writing Coaching Program						
	Total	CAN	NU	SETH	GUMSHOE	P3	STAR
Participants, No (%)	545 (100%)	117 (21%)	112 (21%)	106 (19%)	98 (18%)	67 (12%)	45 (8%)
Female, No. (%)	364 (67%)	68 (58%)	67 (60%)	73 (69%)	77 (79%)	53 (79%)	26 (58%)
URM^a^	331 (61%)	71 (61%)	67 (60%)	70 (66%)	50 (51%)	41 (61%)	32 (71%)
**Race and Ethnicity, No. (%)**							
American Indian/Alaska Native	19 (3%)	1 (1%)	1 (1%)	1 (1%)	14 (14%)	2 (3%)	0 (0%)
Asian	70 (13%)	14 (12%)	14 (13%)	16 (15%)	7 (7%)	12 (18%)	7 (16%)
Black	174 (32%)	37 (32%)	32 (29%)	41 (39%)	19 (19%)	25 (37%)	20 (44%)
Native Hawaiian/Other Pacific Islander	10 (2%)	1 (1%)	0 (0%)	2 (2%)	5 (5%)	2 (3%)	0 (0%)
Hispanic	114 (21%)	30 (26%)	31 (28%)	26 (25%)	6 (6%)	10 (15%)	11 (24%)
White	116 (21%)	28 (24%)	21 (19%)	18 (17%)	34 (35%)	11 (16%)	4 (9%)
>1 race	20 (4%)	3 (3%)	4 (4%)	0 (0%)	9 (9%)	3 (4%)	1 (2%)
Other	7 (1%)	0 (0%)	1 (1%)	2 (2)	2 (2%)	1 (1%)	1 (2%)
Prefer not report	7 (1%)	2 (2%)	2 (2%)	0 (0%)	1 (1%)	1 (1%)	2 (2%)
Missing	8 (1%)	1 (1%)	6 (5%)	0 (0%)	1 (1%)	0 (0%)	0 (0%)
**Career Stage**							
Postdoctoral Fellow	183 (34%)	86 (74%)	34 (30%)	23 (22%)	17 (17%)	12 (18%)	11 (24%)
Instructor	13 (2%)	3 (3%)	3 (3%)	1 (1%)	3 (3%)	3 (4%)	0 (0%)
Assistant Professor	275 (50%)	24 (21%)	61 (54%)	67 (63%)	50 (51%)	46 (69%)	27 (60%)
Other	69 (13%)	1 (1%)	13 (12%)	15 (14%)	27 (28%)	6 (9%)	7 (16%)
Missing	5 (1%)	3 (3%)	1 (1%)	0 (0%)	1 (1%)	0 (0%)	0 (0%)
**Highest Degree Completed**							
PhD or ScD	448 (82%)	95 (81%)	96 (86%)	84 (79%)	81 (83%)	50 (75%)	42 (93%)
MD or DO	39 (7%)	10 (9%)	5 (4%)	8 (8%)	4 (4%)	11 (16%)	1 (2%)
Other Clinical or Professional Doctorate^b^	21 (4%)	2 (2%)	2 (2%)	6 (6%)	9 (9%)	1 (1%)	1 (2%)
MD/PhD	28 (5%)	7 (6%)	7 (6%)	6 (6%)	3 (3%)	4 (6%)	1 (2%)
PharmD/PhD or DVM/PhD	8 (1%)	2 (3%)	1 (2%)	1 (1%)	1 (1%)	1 (1%)	0 (0%)
Master’s degree	1 (0%)	0 (0%)	0 (0%)	1 (1%)	0 (0%)	0 (0%)	0 (0%)

CAN, Committee on Institutional Cooperation Academic Network (University of Chicago); GUMSHOE, Grant writing Uncovered: Maximizing Strategies, Help, Opportunities, and Experiences (University of Colorado Anschutz Medical Campus and Washington State University); NU, Northwestern University Grant Writers Coaching Groups; P3, Proposal Preparation Program (University of Minnesota); SETH, Strategic Empowerment Tailored for Health Equity Investigators (Morehouse School of Medicine); STAR, Steps Towards Academic Research (University of North Texas Health Sciences Center).

^a^ URM, underrepresented minority, is defined as individuals from groups that are underrepresented in biomedical research (https://grants.nih.gov/grants/guide/notice-files/NOT-OD-20-031.html) Data are self-reported and includes participants who indicated belonging to one or more URM racial/ethnic groups.

^b^ AuD, DDS, DrPH, DVM, EdD, JD, PharmD, or PsyD

Participants originated from 187 different institutions and organizations from all regions of the U.S., including Alaska, Hawaii, and Puerto Rico. One hundred and twenty-eight participants (23% of the sample) were from an institution with a particular emphasis on minority populations (i.e. a Historically Black College and University, Hispanic Serving Institution, or Federally designated Minority Serving Institution). Most participants (482, 88%) were affiliated with institutions that had a 2015 Carnegie Classification of either (i) Doctoral University or (ii) Special Focus Four-year Medical or Other Health Professions School. The remainder were affiliated with Master’s Colleges and Universities, Baccalaureate Colleges, or other research institutions without a Carnegie classification.

### Number and distribution of grants awarded

As of March 31, 2019, 164 grants were identified as having been awarded to GCP participants. These awards were distributed across 134 unique participants, some of whom received more than one grant (ranging from 2 to 4). Of the 164 grants, 105 (64%) were awarded to URM investigators and 113 (69%) to female investigators.

**Fig 3:**
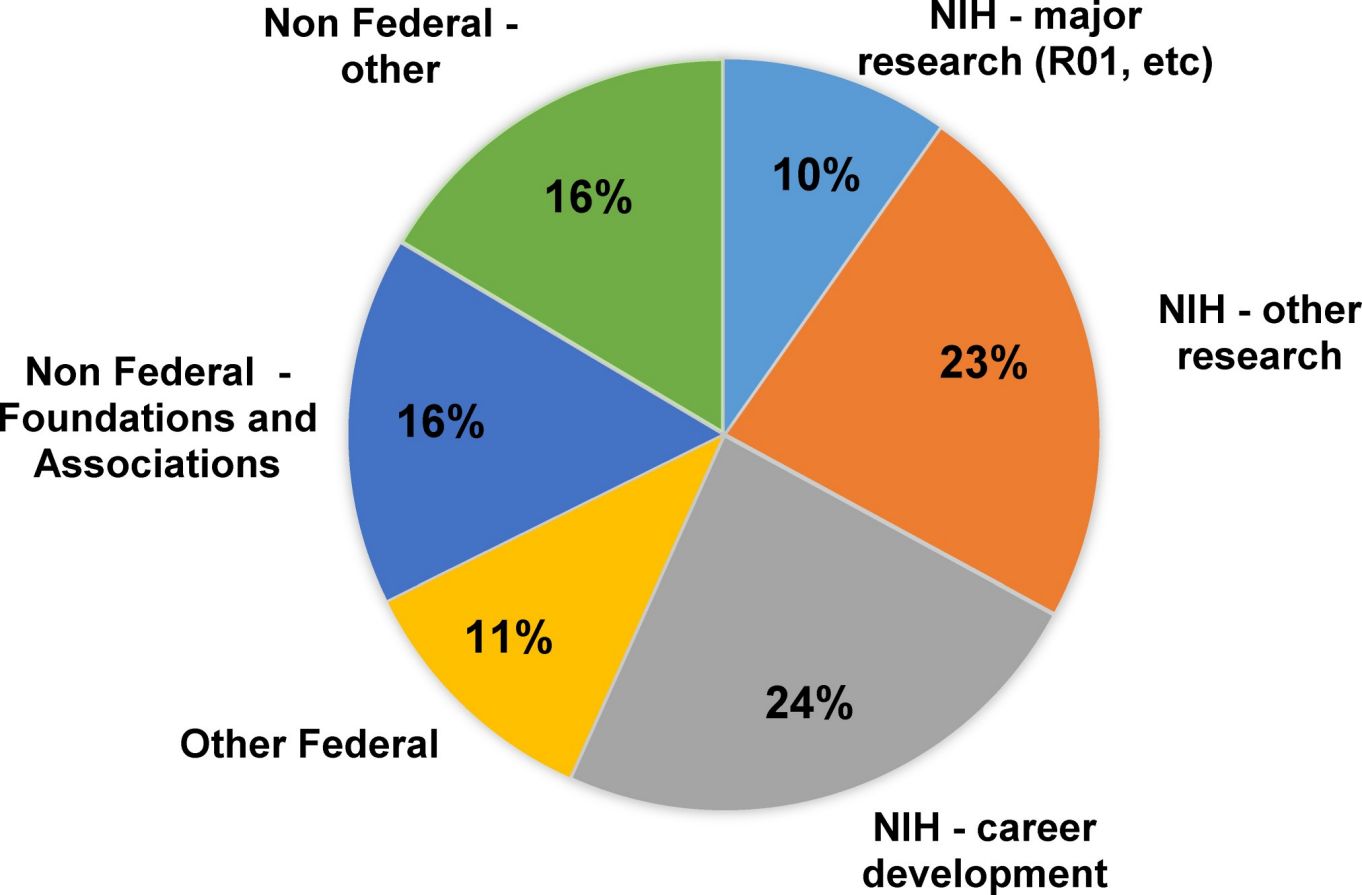
Number (%) of awarded grants for GCP participants by funding category. Data are reported as of March 31, 2019.

Fig 3 presents a breakdown of awarded grants by type of funder. The majority of awards (93, 56%) were from the NIH. These included 16 major NIH grants, defined as R01 awards and similar mechanisms that offer substantial funding and reflect a high level of research independence for investigators; 38 career development grants (F’s, Ks, and Diversity Supplements); and 39 other types of research grants (smaller R awards such as R03s and R21s, SCORE grants; pilot projects funded by parent grants such as P30s or U54s). A more detailed list of awarded NIH grants is presented in Table 2. Over two-thirds of NIH grants were awarded to URM and female investigators. The distribution of awarded grants by NIH institute, center, or office is shown in Table 3.

**Table 2:** Total NIH grants awarded to GCP participants as of March 31, 2019.

Grant Mechanism^a^	Total Number of Awards	Number (%) Awarded to URM Participants^b^	Number (%) Awarded to Female Participants
F31, F32, FI2	6	4 (67%)	6 (100%)
Diversity Supplements	8	8 (100%)	7 (88%)
K01, K08, K22, K23, K12, KL2	24	15 (63%)	17 (71%)
P20, P60	3	3 (100%)	2 (67%)
R01, DP2, U01, U54	16	9 (56%)	12 (75%)
Other R-series (R03, R15, R21, R34, R37, R56)	21	14 (67%)	15 (71%)
SC1, SC2, S06	3	3 (100%)	1 (33%)
Pilot or feasibility from parent NIH grant (such as P30, UL1, U54)	12	9 (75%)	7 (58%)
**Total NIH Grants Awarded**	**93**	**65 (70%)**	**67 (72%)**
**Unduplicated Individuals Receiving NIH Grants**	**80**	**54 (68%)**	**57 (71%)**

^**a**^ Activity codes for NIH grant mechanisms are provided on the NIH website at https://grants.nih.gov/grants/funding/ac_search_results.htm.

^b^ URM, underrepresented minority, is defined as individuals from groups that are underrepresented in biomedical research (https://grants.nih.gov/grants/guide/notice-files/NOT-OD-20-031.html) Data are self-reported and includes participants who indicated belonging to one or more URM racial/ethnic groups.

**Table 3:** Distribution of GCP participants’ awarded NIH grants by institute, center, or office as of March 31, 2019.

NIH Institute, Center, or Office^a^	Number of Grants Awarded to GCP Participants
NCI	12
NIA	11
NCATS	9
NIGMS	8
NINDS	8
NIMHD	7
NICHD	5
NIDA	5
NIDDK	5
NIMH	5
NHLBI	3
NIAID	2
NIBIB	2
NIDCR	2
NIEHS	2
FIC	1
NAIMS	1
NIAAA	1
NIAMS	1
NINR	1
OD	1
Missing data	1
**Total**	**93**

^a^ Abbreviations for specific NIH institutes, centers, and offices are defined at https://www.nih.gov/institutes-nih/list-nih-institutes-centers-offices.

The remaining 71 grants were awarded by other (non-NIH) federal agencies and by non-federal sources (Fig 3). Half of the 18 other federal grants came from the National Science Foundation, with the remainder distributed among six other agencies. The 53 non-federal awards were about evenly distributed between two subcategories: (i) foundation and association grants (e.g., American Heart Association, Robert Wood Johnson Foundation); and (ii) “other,” comprising a mix of mostly state-level grants and institutional awards. A more detailed list of other federal and non-federal grants awarded is presented in Table 4. Approximately two thirds of the other federal grants and half of the non-federal grants were awarded to URM investigators. Female investigators received approximately one third of the other federal grants and three fourths of the non-federal grants.

**Table 4:** Total Other Federal (non-NIH) Grants and Non-Federal Grants Awarded to GCP Participants as of March 31, 2019.

Funding Source	Number of Awards	Number (%) Awarded to URM Participants^a^	Number (%) Awarded to Female Participants
**Other Federal Agencies (excludes the NIH)**			
National Science Foundation	9	5 (56%)	3 (33%)
Veterans Administration	2	1 (50%)	0 (0%)
Patient-Centered Outcomes Research Institute	1	1 (100%)	0 (0%)
Other^b^	6	5 (83%)	3 (50%)
**Total Other Federal Grants Awarded**	**18**	**12 (67%)**	**6 (33%)**
**Unduplicated Individuals Receiving Other Federal Grants**	**16**	**11 (69%)**	**6 (38%)**
**Non-Federal Sources**			
Foundations	17	9 (53%)	14 (82%)
Associations	9	4 (44%)	8 (89%)
Other non-federal sources^c^	27	15 (56%)	19 (70%)
**Total Non-Federal Grants Awarded**	**53**	**28 (52%)**	**41 (77%)**
**Unduplicated Individuals Receiving Non-Federal Grants**	**51**	**26 (51%)**	**40 (78%)**

^a^ URM, underrepresented minority, is defined as individuals from groups that are underrepresented in biomedical research (https://grants.nih.gov/grants/guide/notice-files/NOT-OD-20-031.html) Data are self-reported and includes participants who indicated belonging to one or more URM racial/ethnic groups.

^b^ Includes Department of Defense, National Aeronautics and Space Administration, National Institute of Justice, and United States Department of Agriculture

^c^ Institution, state, other local organizations

### Submission and award rates by application category

**Fig 4:**
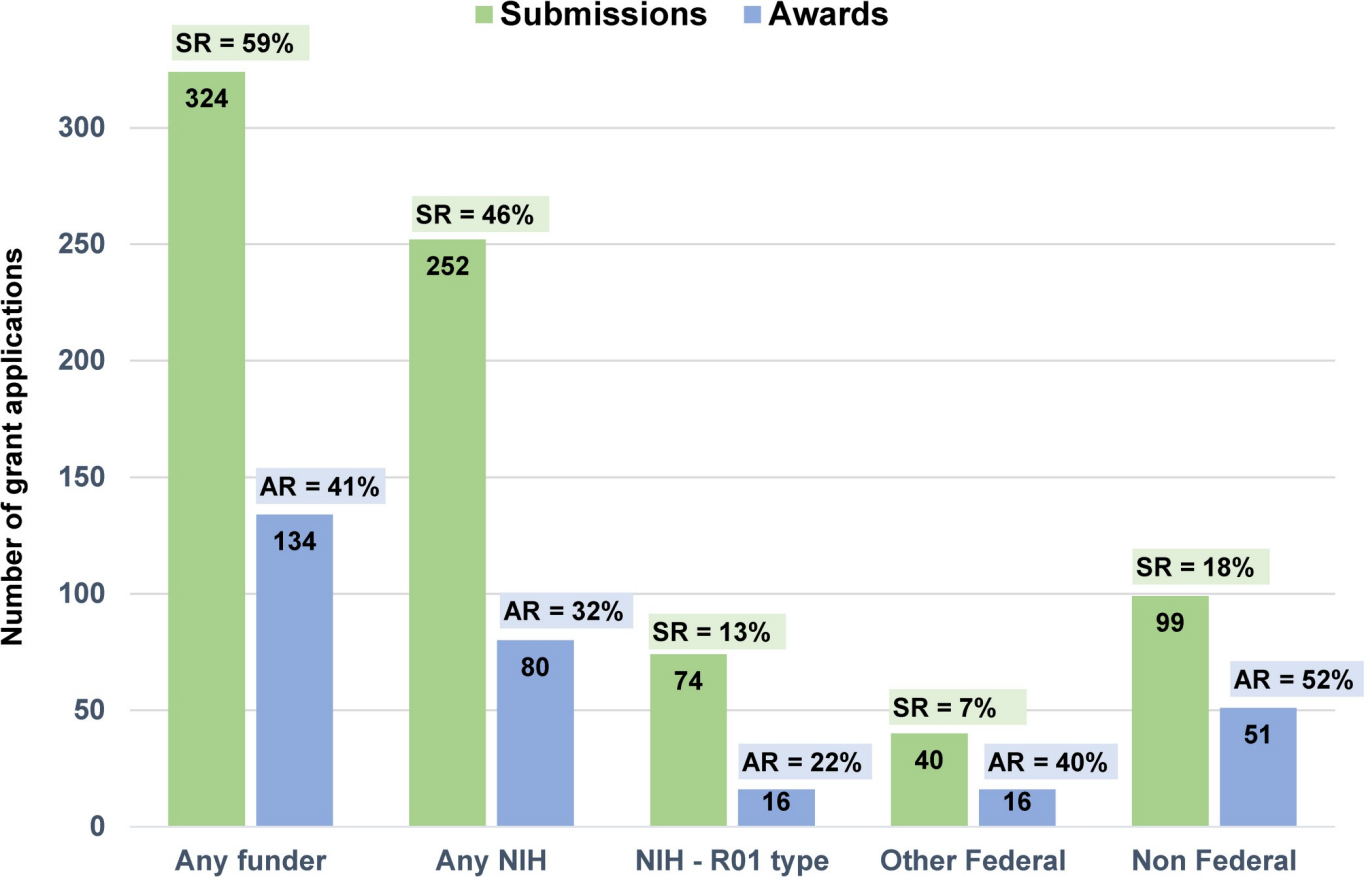
Submission Rates (SR) and Award Rates (AR) across different grant application categories. This is a count of individuals, not proposals, with each individual counted once per category. SR = number of unique participants who submitted/545 total participants. AR = number of unique participants who were funded/number of unique participants who submitted within a category. Data are reported as of March 31, 2019.

In addition to absolute counts of grants awarded, we determined proposal submission rates and award rates for (i) all funding sources and mechanisms combined and (ii) within specific application categories. Fig 4 offers a visual summary of submission and award rates for GCP participants across different types of applications. We derived submission and award rates from counts of individual participants who submitted or were awarded at least one grant proposal in a particular category; they do not reflect multiple submissions or multiple awards attributed to the same person. Our goal for these analyses was to descriptively explore similarities and differences in these two metrics for different types of grants, participant subgroups, and GCP models. We describe these results in more detail below and in Table 5.

**Table 5:** Submission rates and award rates as of March 31, 2019 for the total sample and for participant subgroups by URM status, sex, and model.

Participants	# Trained	All Funders		NIH Any Mechanism		NIH Major Awards		Other Federal		Non-Federal	
		# Who Submitted/ # Trained (%)	# Individuals Funded/ # Who Submitted (%)	# Who Submitted/ # Trained (%)	# Individuals Funded/ # Who Submitted (%)	# Who Submitted/ # Trained (%)	# Individuals Funded/ # Who Submitted (%)	# Who Submitted/ # Trained (%)	# Individuals Funded/ # Who Submitted (%)	# Who Submitted/ # Trained (%)	# Individuals Funded/ # Who Submitted (%)
Total	545	324/545 (59%)	134/324 (41%)	252/545 (46%)	80/252 (32%)	74/545 (13%)	16/74 (22%)	40/545 (7%)	16/40 (40%)	99/545 (18%)	51/99 (52%)
Racial/Ethnic Minority Status											
URM^a^	331	189/331 (57%)	85/189 (45%)	147/331 (44%)	54/147 (37%)	36/331 (11%)	9/36 (25%)	19/331 (6%)	11/19 (58%)	53/331 (16%)	26/53 (49%)
Non-URM	214	135/214 (63%)	49/135 (36%)	105/214 (49%)	26/105 (25%)	38/214 (18%)	7/38 (18%)	21/214 (10%)	5/21 (24%)	46/214 (21%)	25/46 (54%)
Sex											
Female	364	226/364 (62%)	97/226 (43%)	179/364 (49%)	57/179 (32%)	47/364 (13%)	11/47 (23%)	25/364 (7%)	6/25 (24%)	72/364 (20%)	40/72 (56%)
Male	181	98/181 (54%)	37/98 (38%)	73/181 (40%)	23/73 (31%)	27/181 (15%)	5/27 (19%)	15/181 (8%)	10/15 (67%)	27/181 (15%)	11/27 (41%)
Model											
CAN	117	61/117 (52%)	36/61 (59%)	42/117 (36%)	20/42 (48%)	12/117 (10%)	4/12 (33%)	8/117 (7%)	6/8 (75%)	25/117 (21%)	16/25 (64%)
NU	112	63/112 (56%)	23/63 (36%)	50/112 (45%)	15/50 (30%)	13/112 (12%)	1/13 (8%)	10/112 (9%)	4/10 (40%)	18/112 (16%)	5/18 (28%)
SETH	106	56/106 (53%)	20/56 (36%)	47/106 (44%)	14/47 (30%)	13/106 (12%)	3/13 (23%)	7/106 (7%)	2/7 (29%)	8/106 (8%)	5/8 (63%)
GUMSHOE	98	63/98 (64%)	24/63 (38%)	47/98 (48%)	13/47 (28%)	13/98 (13%)	3/13 (23%)	8/98 (8%)	3/8 (38%)	24/98 (24%)	11/24 (46%)
P3	67	52/67 (78%)	24/52 (46%)	45/67 (67%)	15/45 (33%)	18/67 (27%)	5/18 (28%)	5/64 (7%)	1/5 (20%)	16/67 (24%)	10/16 (63%)
STAR	45	29/45 (64%)	7/29 (24%)	21/45 (47%)	3/21 (14%)	5/45 (11%)	0/5 (0%)	2/45 (4%)	0/2 (0%)	8/45 (18%)	4/8 (50%)

CAN, Committee on Institutional Cooperation Academic Network; GUMSHOE, Grant writing Uncovered: Maximizing Strategies, Help, Opportunities, and Experiences (University of Colorado Anschutz Medical Campus and Washington State University); NU, Northwestern University Grant Writers Coaching Groups; P3, Proposal Preparation Program (University of Minnesota); SETH, Strategic Empowerment Tailored for Health Equity Investigators (Morehouse School of Medicine); STAR, Steps Towards Academic Research (University of North Texas Health Sciences Center).

This is a count of individual participants, not proposals. Some participants submitted and were awarded more than one grant, which is not reflected in this table.

^a^ URM, underrepresented minority, is defined as individuals from groups that are underrepresented in biomedical research (https://grants.nih.gov/grants/guide/notice-files/NOT-OD-20-031.html) Data are self-reported and includes participants who indicated belonging to one or more URM racial/ethnic groups.

#### All funding sources

From the total sample, 324 individuals submitted at least one grant application as a principal investigator to any funding agency, yielding an overall submission rate of 59% (Table 5). Of the 324 submitters, 134 investigators (41%) received funding. The observed variability in overall submission rates and funding rates for URM vs. non-URM participants and for men vs women was not statistically significant (p>0.05 by mixed model logistic regression, adjusted for clustering by program and cohort). Across GCP models, submission rates for proposals to any funder ranged from 52% to 78%, and award rates ranged from 24% to 59%.

#### NIH funding mechanisms

Given the origins of NRMN and its mission, we were particularly interested in determining participants’ submission and award rates for grant applications to the NIH. Two hundred and fifty-two individuals (46% of 545) submitted at least one NIH grant application, and 32% of those (80 of the 252) were funded. Once again, the observed variability in NIH submission rates and NIH funding rates for URM vs. non-URM participants and for men vs women was not statistically significant (p>0.05 by mixed model logistic regression, adjusted for clustering by program and cohort). Across GCP models, the submission rate for NIH proposals was highest for P3 (67%), and the award rate for NIH proposals was highest for CAN (48%).

Of the 252 individuals who submitted an NIH grant proposal, 74 applied for a major award (defined above), of which 16 (22%) were funded. Given the small cells sizes for this category, we did not conduct statistical comparisons for different subgroups. Program-specific data suggest that participants in P3 were about twice as likely to submit applications for major NIH awards than participants in other models, and that NU and STAR participants had less funding success in this category than individuals in other programs.

#### Other federal and non-federal funding sources

The last set of results in Table 5 are submission and award rates for applications to federal funding agencies other than the NIH and non-federal funders. Only 7% of participants (40 out of 545) submitted at least one application to a federal agency other than the NIH; however, the overall award rate for this category was high (40%, 16 of 40 individuals funded). For grant applications to non-federal funders, 99 individuals (18%) submitted at least one proposal and just over half of these (52%) received at least one award. This is the highest award rate for the total sample among all grant categories examined.

### Submission and award rates by institution type

Most participants were affiliated with doctoral degree granting universities or other institutions with high levels of research activity; however, the coaching programs also engaged faculty working in other institutional types. The submission and funding rates for participants by type of institution are shown in Table 6.

**Table 6:** Submission rates and award rates as of March 31, 2019 for grant applications to any funder and the NIH, by participant institution type (2015 Carnegie Classification).

2015 Carnegie Classification	# Trained	All Funders		NIH Any Mechanism	
		# Who Submitted/ # Trained (%)	# Individuals Funded/ # Who Submitted (%)	# Who Submitted/ # Trained (%)	# Individuals Funded/ # Who Submitted (%)
Doctoral Universities: Highest Research Activity	317	198/317 (62%)	94/198 (47%)	156/317 (49%)	55/156 (35%)
Doctoral Universities: Higher Research Activity	85	56/85 (66%)	18/56 (32%)	40/85 (47%)	12/40 (30%)
Doctoral Universities: Moderate Research Activity	19	6/19 (32%)	1/6 (17%)	3/19 (16%)	0/3 (0%)
Special Focus Four-Year: Medical Schools & Centers, Other Health Professions Schools	61	34/61 (56%)	10/34 (29%)	31/61 (51%)	7/31 (23%)
Master's Colleges & Universities	33	15/33 (45%)	5/15(33%)	11/33 (33%)	3/11 (27%)
Baccalaureate Colleges	13	8/13 (67%)	3/8 (38%)	4/13 (31%)	0/4 (0%)
Specialized Research Facility^a^	17	7/17 (41%)	3/7 (43%)	7/17 (41%)	3/7 (43%)
**Grand Total**	**545**	**324/545 (59%)**	**134/324 (41%)**	**252/545 (46%)**	**80/252 (32%)**

^**a**^Any institution or organization not included in the 2015 Carnegie Classification dataset

## Discussion

Studies of biomedical research grant writing interventions are scarce in the literature. Published reports typically focus on evaluations of single-institution approaches and/or programs that embed proposal writing activities and mentoring within more comprehensive training models [25–28]. Our results offer promising early outcomes data for an innovative group coaching approach to grant writing that was implemented nationally in six variations, all aimed at the longer term goal of reducing race/ethnicity and gender disparities in NIH proposal (re)submission and award rates.

### Engagement of a diverse national sample

Demographic and institutional data for the 545 NRMN GCP participants support the core premise that productive grant writing coaching groups can be convened successfully beyond institutional boundaries to support investigators from a variety of settings. Sixty-one percent of participants self-identified as belonging to URM groups in biomedical research, and about a quarter were employed at minority serving institutions. Female, Black, and Hispanic investigators were particularly well represented, accounting for 67%, 32%, and 21% of the total sample, respectively. In comparison, a report on the CTSA hubs found that 54% of KL2 awardees were female and only 12% self-identified as underrepresented in biomedical research [29].

We attribute some of our recruitment success to targeted strategies such as SETH’s recruitment within the RCMI network, STAR’s focus on investigators at minority serving institutions, and GUMSHOE’s selection of participants based on a disparity population focus. However, even programs that recruited more broadly had a large proportion of URM participants. We posit that the delivery of the GCPs under the umbrella of the diversity-focused NRMN helped to attract a diverse pool of applicants. While the GPCs were open to URM and non-URM applicants, the recruitment messaging for these programs used language that reflected the overarching goal of the NRMN’s Professional Development Core: To address the unmet need for more diversity in the biomedical research workforce by creating and nationally disseminating transformative, high impact professional development programs to support mentees from diverse backgrounds. Thus, the invitations to participate were welcoming to URM investigators, but not to the exclusion of others.

### Proposal submission rates

Our results indicate that different variations of an intensive coaching intervention can be leveraged to support the grant writing efforts of biomedical researchers at different stages of project development. Nearly 60% of all GCP participants submitted at least one proposal to any funder within 18 months of program completion, and 46% submitted at least one NIH proposal. Although NIH applications were emphasized in the GCPs, applications to other funders were also recognized as valuable, particularly within models that accepted investigators with less grant writing experience.

Ideally, all participants would have reached the submission benchmark, but in reality many needed additional time beyond the GCPs’ duration. It was not uncommon during the coaching process to uncover a need for more preliminary data or even a shift in project direction to strengthen a proposal’s research questions, hypotheses, or approach. Participants who lacked strong support or capacity for research at their home institutions may have struggled to take the necessary next steps to finalize their proposal. Simply finding time could be a barrier for some participants, especially once the accountability structure of the GCP ended. We know that others entered a program too early to derive immediate benefit. SETH program leaders identified this critical issue in their earliest cohorts, prompting them to implement an enhanced screening process (semi-structured interview protocol) to better evaluate applicants’ readiness for writing. This change, coupled with implementation of their online support community, significantly reduced participants’ time to proposal submission [22].

### Grant acquisition and award rates

A laudable 25% of all GCP participants received at least one grant within 18 months or less of program completion. Specifically, 80 of the 545 participants (15%) acquired NIH funding, 16 (3%) other federal funding, and 51 (9%) non-federal funding. In comparison, a report by Sorkness and colleagues documented the extramural research funding success of CTSA scholars within one year of completing a KL2 program [29]. For the 47 CTSA hubs that provided data, a median of 27% of scholars received NIH support within one year of the KL2, 6% received other federal funding, and 12% received foundation funding. The more favorable KL2 outcomes are not unexpected; this difference could be attributed to the high level of competitiveness of the KL2 selection process–in contrast to the more inclusive selection process used by the GCPs–and the intensive 2 to 3 years of mentored research training that all KL2 scholars receive.

Of the 74 major (R01-like) NIH research grants submitted by GCP participants, 16 have been funded thus far (56% to URM, 75% to women). We expect this outcome to increase over time given the long time lag associated with submissions, resubmissions, and funding decisions for these types of proposals. In the short term, we can point to a promising 22% award rate for this grant category among GCP participants (25% for URM). This metric most closely corresponds to NIH success rates, which for new R01 grants was 17.3% for fiscal year 2016, 16.7% for 2017, and 17.8% for 2018 (data retrieved from the NIH Research Portfolio Online Reporting Tools [30]). Among GCP submitters, award rates were lowest for major NIH grants (22%), likely reflecting the competitiveness of this funding source, and highest for non-federal applications (52%), which include grants from potentially more accessible sources such as institutional funds, private foundations, and local or state agencies. Funding rates for the many possible non-federal sources reflected in our dataset are not readily available.

Over 80% of the NIH grants awarded to GCP participants were for mechanisms that supported mentored research training experiences or other shorter term, smaller scope projects. Grants such as these, whether from the NIH or other funders, are critical for preparing the pipeline of early-career URM and female investigators who can later pursue and be competitive for larger, R01-type awards as they achieve independence and demonstrate productivity.

The majority of grant recipients in our sample were URM and/or female. We cannot draw conclusions about observed subgroup differences in award rates (by sex, URM status, degree, career stage) given the small cell sizes for some categories and potential for uncontrolled confounding and/or selection bias on factors that might influence this outcome (such as scientific topic area, specific type of grant, and particular funding agency/NIH institute targeted by the applicant). Nonetheless, our findings offer strong preliminary support that GCPs are an effective intervention for helping postdoctoral and faculty participants, including URM and women, to acquire biomedical research funding from a range of funders.

### Overall value of the GCP approach

Although this report focuses on near term proposal outcomes, there is inherent value in providing rigorous grant writing coaching to a large, heterogeneous group of investigators–many of whom lacked strong local mentorship and a robust understanding of the grant writing genre–who are now better equipped for long term success. Indicative of this, in an earlier analysis of the first 190 GCP completers we found that participants reported post-training self-efficacy gains in three domains: conceptualizing a study, designing a study, and acquiring funding for a study [31]. We intentionally did not focus the GCP solely on investigators at the cusp of grant-getting success or working in well-resourced, highly research-intensive environments, which might have yielded higher short-term success rates. Rather, we sought to fill gaps in mentoring (particularly among URM), enrich the pipeline to research independence via acquisition of pilot and training grants, and support the stepwise progression of investigators in different institutional settings and at different stages of professional development. Consequently, for many participants the benefits of GCP participation will only be realized several months and even years into the future, requiring longer term follow up. Our results suggest that the GCP approach can be successful across a large spectrum of institution types, including those that place greater emphasis on their teaching mission. An important role of GCP coaches was helping participants to identify appropriate funding mechanisms and take advantage of grants available specifically to researchers within their type of institution.

### Differences across GCP models

Our rationale for testing six different GCP models in phase I of NRMN was twofold: first, to offer a variety of training programs for investigators at different career stages and experience levels (congruent with NRMN’s mentoring mission); second, to acquire preliminary data about each model’s effectiveness–knowledge that could be applied to design a more optimized intervention for future testing in a more rigorous trial design. In the absence of study design features such as randomization and strict inclusion/exclusion criteria for participants, we must be cautious in drawing conclusions about the relative effectiveness of the different models. For this phase of the NRMN GCP initiative, we limit our cross-model comparisons to some preliminary interpretations, acknowledging that they will need additional testing to support.

Some of the apparent between-model differences appear logically related to their unique design features. For example, P3 participants had the highest NIH submission rate, which aligns with that model’s stricter selection of applicants who were developing K- or R-series proposals, very clearly ready to write, and planning to submit soon after program completion. Other models applied broader selection criteria, placing more emphasis on an applicant’s need for coaching and welcoming greater variety in the types of proposals being developed. Differences in award rates, too, might be due to model characteristics. For example, CAN participants achieved the highest overall award rate. This might be attributed to a combination of target audience (CAN had the highest number of postdoctoral fellows, and awards for this career stage typically have higher rates of funding than faculty-level awards) and program design (after the kickoff, CAN coaching sessions were held in-person on local campuses, allowing the input of multidisciplinary coaches on participants’ proposals). In the NU model, groups typically only worked through the Specific Aims, Significance, and Innovation sections of NIH grants (plus Candidate and Career Development sections for K awards). When this model is conducted at Northwestern University, all participants have substantial scientific input from colleagues and/or or mentors in their fields to help them develop their Approach sections. However, as applied in this national model, NU appeared to perform slightly less well than some others, especially for major NIH awards, suggesting that this “lighter touch” model may be optimal only when individuals have strong scientific preparation and/or support systems in their home institutions.

### Limitations, conclusions, and future work

Our submission and award counts may be underestimates, as they are largely based on self-report from follow up surveys conducted through 18 months after program completion. Response rates to our surveys were quite high for surveys in general (ranging from 87% right after the programs to 55% at 18 months later), but we expect that at least some of the non-responders submitted proposals and were awarded grants that are not represented in our results.

As noted above, given the intentional differences in GCP models and their implementation as pilot programs during NRMN phase 1, we must be cautious in drawing conclusions about their impact relative to one another or to other interventions. However, our data support the feasibility of implementing productive intra- and inter-institutional grant writing coaching groups to support early-career investigators, including URM and those who may not have strong local mentorship. Moreover, our results provide compelling preliminary evidence that this intervention approach–engaging participants in several months of sustained, intensive coaching with experienced investigators, nested within a small cohort of peers all actively writing grant applications–can help participants to achieve the important benchmarks of proposal submission and funding.

Building on our phase 1 results, several members of our team have received NRMN phase 2 funding (NIH U01 grants) to more rigorously study GCP interventions, using randomized trial designs and mixed methods analytic approaches. These studies are designed to identify specific features of the group coaching approach that influence its effectiveness and to determine which participants are more or less likely to benefit from this type of intervention.

## Supporting information

S1 Fig Sample schedule for P3 model in the NRMN Grant Writing Coaching Program. (PDF)

S1 Dataset (XLS)
