# Supplementary material for: Grant application outcomes for biomedical researchers who participated in the National Research Mentoring Network’s Grant Writing Coaching Programs
Source: PLoS One. 2020 Nov 9;15(11):e0241851. doi: 10.1371/journal.pone.0241851 (PMC7652313; doi:10.1371/journal.pone.0241851)
Supplement: S1 Fig — (PDF) [file pone.0241851.s001.pdf]

Pre-Program Activities

- Writing assignment: Due January 3, 2018
  - Updated *Specific Aims* page and *Biosketch*
- Review video of rhetorical patterns and *Specific Aims* examples from successful proposals

Kickoff @ U of MN Minneapolis campus: January 8-9, 2018

- Introduction to NRMN-P^3
- Review of participants' *Specific Aims* and *Biosketches*
- Early-stage investigator panel
- Document design principles
- Individual meetings with coaches
- Discussion with NIH officials
- Prep for future sessions

Virtual meeting 1: January 25, 2018, 1-3pm CST

- Writing assignment: Due January 22, 2018
  - Revised *Aims + Significance*

Virtual meeting 2: February 8, 2018 1-3pm CST

- Writing assignment: Due February 5, 2018
  - R's: Revised draft-to-date + outline of *Approach*
  - K's (additional): *Candidate Background*

Virtual meeting 3: March 1, 20181-3pm CST

- Writing assignment: Due February 26, 2018
  - R's: Revised draft-to-date + *Innovation* + *Preliminary Work* section of *Approach*
  - K's (additional): Revised *Candidate Background* + *Career Goals & Objectives*

Virtual meeting 4: March 15, 2018 1-3pm CST

- Writing assignment: Due March 12, 2018
  - R's: Revised draft-to-date + expanded *Approach* (building from outline)
  - K's (additional): Revised *Career Goals & Objectives + Training Activities*

Virtual meeting 5: March 29, 2018 1-3pm CST

- Writing assignment: Due March 26, 2018
  - R's: Revised draft-to-date + expanded *Approach*
  - K's (additional): Revised *Training Activities + Training in RCR*

Virtual meeting 6: April 12, 2018, 1-3pm CST

- Writing assignment: Due April 9, 2018
  - R's: Revised draft-to-date + expanded *Approach,* must include *Data Analysis* and brief paragraph(s) on *Potential Problems and Alternative Approaches*
  - K's (additional): Revised *Training in RCR*

Virtual meeting 7: April 26, 2018 1-3pm CST

- Writing assignment: Due April 23, 2018
  - R's and K's: All prior sections revised
  - Revised *Biosketch*
  - Optional to submit other sections

Mock Study Section @ U of MN Minneapolis campus: May 15, 2018 (reviewers call in)

- Writing assignment: Final draft for Practice Study Section, Due May 4, 2018
  - <u>Required</u>: PI Biosketch, Specific Aims, Research Strategy, K-specific sections completed during P3
  - <u>Optional</u>: Budget and Justification, Resources, Human Subjects/Vertebrate Animals, Environment and Institutional Commitment to Candidate (K's), etc.
- Individual meetings with coaches

Post-Program Activities

- Participant follow-up phone call with mock reviewer as needed
- Individualized support from coaches as needed
